# Supplementary material for: The impact of phage and phage resistance on microbial community dynamics
Source: PLoS Biol. 2024 Apr 22;22(4):e3002346. doi: 10.1371/journal.pbio.3002346 (PMC11034675; doi:10.1371/journal.pbio.3002346)
Supplement: S1 Text — (PDF) [file pbio.3002346.s001.pdf]

Text S1

1 Community model parameterization

We utilize the generalized Lotka-Volterra (gLV) competition model framework and least-squares regression in order to parameterize growth and interaction coefficients for four bacterial species using time series data in line with other microbiome studies [1–5]. We define the gLV model

$$\frac{dN_i(t)}{dt} = r_i N_i(t) - \sum_{j=1}^{n} \beta_{ij} N_i(t) N_j(t) \tag{S.1}$$

where $N_i(t)$ is the density of species i at time t and r_i is the corresponding maximal growth rate ($r_i > 0$, $\forall i = 1, ..., n$). The interaction coefficients $\beta_{i,j}$ describe the per-capita inhibitory effect of species j on species i. We assume competitive interactions, $\beta_{ij} > 0$, $\forall i, j = 1, ..., n$. For all models, we scale densities N_i by the maximum density of species i for fitting and then adjust coefficients for this scaling after optimal parameters have been selected.

1.1 Single species models

Initial fitting of single species maximal growth rates was done using single species optical density curves (OD_{600}) in time (hrs) calibrated to cfu/ml. Model fitting was done using least squares regression and the logistic growth equation

$$\frac{dN_i(t)}{dt} = r_i N_i(t) \left(1 - \frac{N_i(t)}{k_i}\right) \tag{S.2}$$

which corresponds to the single species version of Equation S.1 where $\beta_{ii} = r_i/k_i$ and k_i describes the single species carrying capacity. Fitting was done by solving the ordinary differential equation (ODE) defined in Equation S.2 using `ode45` in Matlab for $\tilde{N}_i(t)$ and iteratively minimizing the sum of squared errors (SSE), $\sum(N_i - \tilde{N}_i)^2$, via the Levenberg-Marquardt algorithm. The maximal growth rates r_i were then fixed from these parameterizations for fitting all multi-species models.

1.2 Multi-species models

In contrast to the single-species case where we fit the solution of the ODE, here we linearize the equations for model fitting, using the mathematical definition $\frac{d\ln N_i}{dt} = \frac{1}{N_i}\frac{dN_i}{dt}$, and fit the natural log derivative of $N_i(t)$, as is commonly done in microbiome modeling [1–3, 6, 7]. Rewriting Equation S.1 as

$$\frac{d\ln N_i}{dt} = r_i - \sum_{j=1}^{n} \beta_{ij} N_j \tag{S.3}$$

we approximate the natural log derivative of N_i using either the `gradient` function in Matlab or via spline fitting [3]. From here we solve the least-squares problem iteratively minimizing the SSE between the data approximated log derivative $\frac{d\ln N_i}{dt}$ and the model calculated log derivative $\frac{d\ln \tilde{N}_i}{dt}$ using the Levenberg-Marquardt algorithm, and also via code from [3].

The resulting models were evaluated using the coefficient of determination,

$$R^2 = 1 - \frac{\sum_{i=1}^{M}(y_i - \hat{y}_i)^2}{\sum_{i=1}^{M}(y_i - \bar{y}_i)^2} \tag{S.4}$$

where y is the data, $\bar{y}$ is the mean of the data, $\hat{y}$ is the model prediction, M is the total number of time points. This gives us a metric of how well the model predicts the dynamics as compared to the mean of the data. We select the parameterization that gives us the highest R^2 (want R^2 closest to 1). We note that low or negative R^2 values do not indicate that the model doesn't fit the data, just that the mean of the data points over time better captures the dynamics than the model fit.

We constrained parameters for multi-species model fitting in three different ways described below (Sections 1.2.1-3).

1.2.1 Pairwise data constrained

We initially fit multi-species models using a 'bottom-up' approach [6], where we fit all possible pairwise interactions using co-culture experimental data for all pairs, and use the resulting interaction matrix B,

$$B = \begin{bmatrix} \beta_{11} & \dots & \beta_{1n} \\ \vdots & \ddots & \vdots \\ \beta_{n1} & \dots & \beta_{nn} \end{bmatrix}$$

to predict the dynamics of 3- and 4-species communities ($n = 3$ and $n = 4$, respectively). For these fits, single species growth rates r_i were fixed from Section 1.1 and all interaction coefficients $\beta_{i,j}$ were left open. This was done for (1) the WT strain of *P. aeruginosa* (PA14) without phage; (2) the WT strain of *P. aeruginosa* (PA14) with phage where the phage dynamics are not explicitly tracked but implied by allowing interaction coefficients to differ from the corresponding no phage case, (1); (3) the CRISPR-Cas knockout strain (CRISPR-KO) of *P. aeruginosa* without phage; and (4) the CRISPR-Cas knockout strain (CRISPR-KO) of *P. aeruginosa* with phage where again the phage dynamics are not explicitly tracked but implied by allowing interaction coefficients to differ from the corresponding no phage case, (3). We define the resulting full community interaction matrices: (1) B_{PA14}, (2) $B_{PA14,phage}$, (3) $B_{CRISPR-KO}$, (4) $B_{CRISPR-KO,phage}$. Figure S6 depicts the model vs. data for the no phage case, when our growth and interaction terms are derived from only 1- and 2-species experimental data.

1.2.2 Two- and three-species data constrained

We looked to improve the model accuracy by relaxing the dependence on solely pairwise data. To do this, we followed the same procedure described above, but instead of fitting only all possible co-culture combinations, we additionally fit all pairwise interaction coefficients $\beta_{i,j}$ for 3-species experimental data where only single species growth rates r_i were fixed and all interaction coefficients $\beta_{i,j}$ were left open. Here, we don't explicitly include higher order interactions (ex. $\beta_{i,j,k}N_iN_jN_k$), but by leaving the terms open in the 3-species case, we can construct two different matrices B—one built from 2-species data B_{2sp} and one built from 3-species data B_{3sp}. In the absence of higher order interactions, we would assume $B_{2sp} = B_{3sp}$. This is not the case here, in line with our conclusions that the gLV model misses key components of the community and phage dynamics observed experimentally.

We then constructed a new version of the B matrices in Section 1.2.1 by averaging the corresponding interaction coefficients from the 3-species data fits ($\beta_{i,j}$ from fitting the gLV to data from experimental treatments: PA+AB+SA, PA+BC+SA, and PA+BC+AB for PA14 and CRISPR-KO in the presence/absence of phage). For example, if PA = N_1 and AB = N_2, any model for an experiment that includes PA and AB as 2 of the 3 species will give us values for $\beta_{1,1}$, $\beta_{1,2}$, $\beta_{2,1}$, and $\beta_{2,2}$. We assume that by averaging these values from multiple 3-species experiments, we can better capture a combination of implicit higher order interactions (i.e. the average of $\beta_{1,2}$ from PA+AB+SA and $\beta_{1,2}$ from PA+BC+AB will approximate $\beta_{1,2}$ in the full 4-species community better than the value obtained from PA+AB co-culture data). In the absence of phage, this matrix construction performs poorly for the WT strain, so we additionally include all pairwise

data interaction terms from Section 1.2.1 in our averaging. For the CRISPR-KO strain without phage, this combination produces the highest R^2 value of all combinations tested. In the presence of phage, we note that other ways of averaging parameters lead to different qualitative or quantitative model results (some with slightly higher R^2 values), but the general inability of the gLV model to capture the 4-species dynamics from 2- and 3-species data in the presence of phage is consistent ($R^2 < 0$). The chosen parameterization captures the experimentally observed qualitative behavior of the community in the presence of phage (Fig S7).

Figures 9 and 10 show the results of this model parameterization and 4-species prediction, where interaction coefficients corresponding to the WT PA14 case in Figure 9 are shown in Figure S8. Figure S7 shows the corresponding long-time behavior of the models.

1.2.3 Four-species modeling

We also fit a model for the full 4-species community data where only single species growth rates r_i were fixed and all pairwise interaction coefficients $\beta_{i,j}$ were left open. Following from 3-species data fitting, we don't explicitly include higher order terms, but instead imply higher order interactions by allowing the pairwise coefficients $\beta_{i,j}$ to be tuned to the full community data. This produced improved quantitative results in some cases, but struggled with local minima and the number of open parameters in others.

1.2.4 Modeling phage impacts

We chose to model the impact of the phage implicitly in order to limit model complexity with respect to functional forms and the number of parameters. Modelling the community in the absence of phage highlights that the gLV model misses a number of complexities present in the community (i.e. higher order interactions) that are critical for recapitulating the quantitative behavior of the community. Additionally, we find that increasing the number of open parameters (such as in Section 1.2.3 above), leads to issues with local minima given the resolution of our dataset. We briefly explored modeling phage dynamics explicitly, but we found that increases in model complexity and parameter number further exacerbated issues we experienced in the no phage case, without providing justified improvements in model predictive performance. Our use of a simplified model (implicit phage effects and implicit higher-order interactions) allows us to predict qualitative community behavior in both the presence and absence of phage and illustrates the need for critical evaluation of model forms used for quantifying microbiome dynamics in the future.

1.3 Long time simulations

In order to assess long time behavior of the resulting 4-species community model, we simulated the parameterized Equation S.1 for 40 hrs for each experimental treatment (PA WT without phage, PA WT with phage, PA CRISPR-KO without phage, PA CRISPR-KO with phage). These results are shown in Figure S7. We use growth and interaction parameters from the two- and three-species data constrained model fitting (Section 1.2.2, Fig 9, 10, and S8). Simulation was done using `glv_simulation.m` from [3]. We choose the initial population densities where *P. aeruginosa* is either (i) common, using the experimental initial conditions for all species; or (ii) rare, using 10^3 cfu/ml as the initial density for *P. aeruginosa* and experimental initial conditions for all other species. This variation in initial condition did not impact the qualitative outcome of the simulations.

2 Competitive release of *A. baumannii* with phage predation

Here, we consider a model of 2-species competition between a focal and non-focal pathogen in the presence of phage targeting the focal pathogen. We confirm that in the presence of phage, competitive release of the non-focal pathogen will occur when it inhibits the focal pathogen.

We define a simple model of a two species *P. aeruginosa* (focal pathogen) and *A. baumannii* (non-focal pathogen) competitive interaction plus phage, where only *P. aeruginosa* interacts with the phage. We define the variables $P = P(t)$ and $A = A(t)$ to describe the densities in time of *P. aeruginosa* and *A. baumannii*, respectively and denote the phage V. For simplicity and to isolate the effect of phage on qualitative outcomes of two species competition between the alternate dominant species in the presence/absence of phage observed experimentally, we don't allow the phage density to change in time for this example. Following from Equation S.1 and [8], we define the model,

$$\frac{dP}{dt} = r_p P - \beta_{pp} P^2 - \beta_{pa} PA - cPV \quad \text{(S.5a)}$$

$$\frac{dA}{dt} = r_a A - \beta_{ap} AP - \beta_{aa} A^2 \quad \text{(S.5b)}$$

where the definitions for r_i and β_{ij} $(\forall i, j = p, a)$ follow from Equation S.1 and c is the growth cost imposed by the phage on P.

We can determine the equilibria (P^*, A^*) of the system by setting $\frac{dP}{dt} = 0$ and $\frac{dA}{dt} = 0$ simultaneously. We

focus on the case were we have equilibrium coexistence of P and A ($P^*, A^* > 0$), which occurs at

$$(P^*, A^*) = \left(\frac{-(\beta_{pa} r_a - \beta_{aa} r_p + \beta_{aa} cV)}{\beta_{pp}\beta_{aa} - \beta_{pa}\beta_{ap}}, \frac{\beta_{pp} r_a - \beta_{ap} r_p + \beta_{ap} cV}{\beta_{pp}\beta_{aa} - \beta_{pa}\beta_{ap}} \right) \tag{S.6}$$

Assuming that coexistence is stable (Section 2.1), we can define

$$\frac{\partial P^*}{\partial V} = \frac{-c\beta_{aa}}{\beta_{pp}\beta_{aa} - \beta_{pa}\beta_{ap}} \tag{S.7}$$

and

$$\frac{\partial A^*}{\partial V} = \frac{c\beta_{ap}}{\beta_{pp}\beta_{aa} - \beta_{pa}\beta_{ap}} \tag{S.8}$$

describing change in P^* and A^* with change in the phage density. Following from [8], and given that the two species exhibit a mutually inhibitory interaction, we can define a condition for competitive release of *A. baumannii* by solving $\partial A^*/\partial V > 0$, i.e. A^* increasing with V increasing,

$$\frac{\partial A^*}{\partial V} > 0 \tag{S.9}$$

$$\frac{c\beta_{ap}}{\beta_{pp}\beta_{aa} - \beta_{pa}\beta_{ap}} > 0$$

Given stability of the coexistence equilibrium (Section 2.1), we have $\beta_{pp}\beta_{aa} - \beta_{pa}\beta_{ap} > 0$, and the condition for competitive release of A is,

$$c\beta_{ap} > 0 \tag{S.10}$$

meaning that competitive release of *A. baumannii* will always occur when A inhibits P ($\beta_{ap} > 0$) as long as there is an additional cost to *P. aeruginosa* due to the presence of phage ($c > 0$). This is consistent with our results in the main text.

2.1 Constraints for stable coexistence

We define the constraints for stability of the coexistence equilibrium (Equation S.6), such that both populations are nonnegative for biological relevance (can't have a negative population density).

First, we address biological relevance, finding the conditions for $P^*, A^* > 0$.

$$P^* = \frac{-(\beta_{pa} r_a - \beta_{aa} r_p + \beta_{aa} cV)}{\beta_{pp}\beta_{aa} - \beta_{pa}\beta_{ap}} > 0$$

and

$$A^* = \frac{\beta_{pp} r_a - \beta_{ap} r_p + \beta_{ap} cV}{\beta_{pp}\beta_{aa} - \beta_{pa}\beta_{ap}} > 0$$

We address two possible cases: (1) $\beta_{pp}\beta_{aa} - \beta_{pa}\beta_{ap} > 0$ and (2) $\beta_{pp}\beta_{aa} - \beta_{pa}\beta_{ap} < 0$.

Case 1. Given $\beta_{pp}\beta_{aa} - \beta_{pa}\beta_{ap} > 0$, we have

$$\frac{\beta_{pa}}{\beta_{aa}} < \frac{r_p - cV}{r_a} \text{ for } P^* > 0 \tag{S.11}$$

and

$$\frac{\beta_{pp}}{\beta_{ap}} > \frac{r_p - cV}{r_a} \text{ for } A^* > 0 \tag{S.12}$$

Finding the Jacobian of Equation S.5 and evaluating it at Equation S.6, $J_{coexist}$, we evaluate $tr(J_{coexist}) < 0$ and $det(J_{coexist}) > 0$ for stability. Given our assumption that $\beta_{pp}\beta_{aa} - \beta_{pa}\beta_{ap} > 0$,

$$tr(J_{coexist}) = \frac{r_a(\beta_{pp}\beta_{pa} - \beta_{pp}\beta_{aa}) + r_p(\beta_{ap}\beta_{aa} - \beta_{pp}\beta_{aa}) + cV(\beta_{pp}\beta_{aa} - \beta_{ap}\beta_{aa})}{\beta_{pp}\beta_{aa} - \beta_{pa}\beta_{ap}} < 0 \tag{S.13}$$

simplifies to the condition

$$\frac{r_p - cV}{r_a} < \frac{\beta_{pp}(\beta_{aa} - \beta_{pa})}{\beta_{aa}(\beta_{ap} - \beta_{pp})} \text{ if } \beta_{ap} > \beta_{pp} \tag{S.14a}$$

$$\frac{r_p - cV}{r_a} > \frac{\beta_{pp}(\beta_{aa} - \beta_{pa})}{\beta_{aa}(\beta_{ap} - \beta_{pp})} \text{ if } \beta_{ap} < \beta_{pp} \tag{S.14b}$$

Looking at the second stability condition, $det(J_{coexist}) > 0$,

$$det(J_{coexist}) = \frac{-(\beta_{pp} r_a - \beta_{ap}(r_p - cV))(\beta_{pa} r_a - \beta_{aa}(r_p - cV))}{\beta_{pp}\beta_{aa} - \beta_{pa}\beta_{ap}} > 0 \tag{S.15}$$

we find the condition is satisfied if we satisfy the conditions in Equations S.11 and S.12. Thus, for stability of the coexistence equilibrium (Equation S.6) given $\beta_{pp}\beta_{aa} - \beta_{pa}\beta_{ap} > 0$, we must satisfy Equations S.11, S.12, and S.14.

Case 2. Given $\beta_{pp}\beta_{aa} - \beta_{pa}\beta_{ap} < 0$, we have

$$\frac{\beta_{pa}}{\beta_{aa}} > \frac{r_p - cV}{r_a} \text{ for } P^* > 0 \tag{S.16}$$

and

$$\frac{\beta_{pp}}{\beta_{ap}} < \frac{r_p - cV}{r_a} \text{ for } A^* > 0 \tag{S.17}$$

Using the same conditions for stability as defined above in Case 1, Equation S.13 simplifies to

$$\frac{r_p - cV}{r_a} > \frac{\beta_{pp}(\beta_{aa} - \beta_{pa})}{\beta_{aa}(\beta_{ap} - \beta_{pp})} \text{ if } \beta_{ap} > \beta_{pp} \tag{S.18a}$$

$$\frac{r_p - cV}{r_a} < \frac{\beta_{pp}(\beta_{aa} - \beta_{pa})}{\beta_{aa}(\beta_{ap} - \beta_{pp})} \text{ if } \beta_{ap} < \beta_{pp} \tag{S.18b}$$

Equation S.15 simplifies to either the condition

$$\frac{\beta_{pp}}{\beta_{ap}} > \frac{r_p - cV}{r_a} \text{ and } \frac{\beta_{pa}}{\beta_{aa}} > \frac{r_p - cV}{r_a} \tag{S.19a}$$

or the condition

$$\frac{\beta_{pp}}{\beta_{ap}} < \frac{r_p - cV}{r_a} \text{ and } \frac{\beta_{pa}}{\beta_{aa}} < \frac{r_p - cV}{r_a} \tag{S.19b}$$

neither of which are possible given the conditions on $P^*, A^* > 0$ defined by Equations S.16 and S.17. Thus, in this case, nonnegative coexistence is never stable.

References

[1] Richard R. Stein et al. "Ecological Modeling from Time-Series Inference: Insight into Dynamics and Stability of Intestinal Microbiota". In: *PLOS Computational Biology* 9.12 (Dec. 2013), pp. 1–11. DOI: 10.1371/journal.pcbi.1003388. URL: https://doi.org/10.1371/journal.pcbi.1003388.

[2] Vanni Bucci et al. "MDSINE: Microbial Dynamical Systems INference Engine for microbiome time-series analyses". In: *Genome Biology* 17 (June 2016). DOI: 10.1186/s13059-016-0980-6.

[3] Chen Liao, Joao Xavier, and Zhenduo Zhu. "Enhanced inference of ecological networks by parameterizing ensembles of population dynamics models constrained with prior knowledge". In: *BMC Ecology* 20 (Jan. 2020). DOI: 10.1186/s12898-019-0272-6.

[4] Bhusan K. Kuntal, Chetan Gadgil, and Sharmila S. Mande. "Web-gLV: A Web Based Platform for Lotka-Volterra Based Modeling and Simulation of Microbial Populations". In: *Frontiers in Microbiology*

10 (Feb. 2019), p. 288. ISSN: 1664-302X. DOI: 10.3389/fmicb.2019.00288. URL: https://www.frontiersin.org/articles/10.3389/fmicb.2019.00288/full (visited on 11/20/2023).

[5] Charles K. Fisher and Pankaj Mehta. “Identifying Keystone Species in the Human Gut Microbiome from Metagenomic Timeseries Using Sparse Linear Regression”. en. In: *PLoS ONE* 9.7 (July 2014). Ed. by Bryan A. White, e102451. ISSN: 1932-6203. DOI: 10.1371/journal.pone.0102451. URL: https://dx.plos.org/10.1371/journal.pone.0102451 (visited on 11/20/2023).

[6] Jacob D. Davis et al. “Methods of quantifying interactions among populations using Lotka-Volterra models”. In: *Frontiers in Systems Biology* 2 (2022). ISSN: 2674-0702. DOI: 10.3389/fsysb.2022.1021897. URL: https://www.frontiersin.org/articles/10.3389/fsysb.2022.1021897.

[7] Jérôme Mounier et al. “Microbial Interactions within a Cheese Microbial Community”. In: *Applied and Environmental Microbiology* 74.1 (2008), pp. 172–181. DOI: 10.1128/AEM.01338-07. eprint: https://journals.asm.org/doi/pdf/10.1128/aem.01338-07. URL: https://journals.asm.org/doi/abs/10.1128/aem.01338-07.

[8] Kristofer Wollein Waldetoft et al. “Defining the Benefits of Antibiotic Resistance in Commensals and the Scope for Resistance Optimization”. In: *mBio* 14.1 (2023), e01349–22. DOI: 10.1128/mbio.01349-22. eprint: https://journals.asm.org/doi/pdf/10.1128/mbio.01349-22. URL: https://journals.asm.org/doi/abs/10.1128/mbio.01349-22.
